# Discovery and Characterization of BlsE, a Radical *S*-Adenosyl-L-methionine Decarboxylase Involved in the Blasticidin S Biosynthetic Pathway

**DOI:** 10.1371/journal.pone.0068545

**Published:** 2013-07-18

**Authors:** Jun Feng, Jun Wu, Nan Dai, Shuangjun Lin, H. Howard Xu, Zixin Deng, Xinyi He

**Affiliations:** 1 State Key Laboratory of Microbial Metabolism and School of Life Sciences and Biotechnology, Shanghai Jiao Tong University, Shanghai, China; 2 New England Biolabs, Inc., Research Department, Ipswich, Massachusetts, United States of America; 3 Department of Biological Sciences, California State University Los Angeles, Los Angeles, California, United States of America; University of Nottingham, United Kingdom

## Abstract

BlsE, a predicted radical *S*-adenosyl-L-methionine (SAM) protein, was anaerobically purified and reconstituted *in vitro* to study its function in the blasticidin S biosynthetic pathway. The putative role of BlsE was elucidated based on bioinformatics analysis, genetic inactivation and biochemical characterization. Biochemical results showed that BlsE is a SAM-dependent radical enzyme that utilizes cytosylglucuronic acid, the accumulated intermediate metabolite in *blsE* mutant, as substrate and catalyzes decarboxylation at the C5 position of the glucoside residue to yield cytosylarabinopyranose. Additionally, we report the purification and reconstitution of BlsE, characterization of its [4Fe–4S] cluster using UV-vis and electron paramagnetic resonance (EPR) spectroscopic analysis, and investigation of the ability of flavodoxin (Fld), flavodoxin reductase (Fpr) and NADPH to reduce the [4Fe–4S]^2+^ cluster. Mutagenesis studies demonstrated that Cys_31_, Cys_35,_ Cys_38_ in the C×××C×MC motif and Gly_73_, Gly_74_, Glu_75_, Pro_76_ in the GGEP motif were crucial amino acids for BlsE activity while mutation of Met_37_ had little effect on its function. Our results indicate that BlsE represents a typical [4Fe–4S]-containing radical SAM enzyme and it catalyzes decarboxylation in blasticidin S biosynthesis.

## Introduction

**Figure 1:**
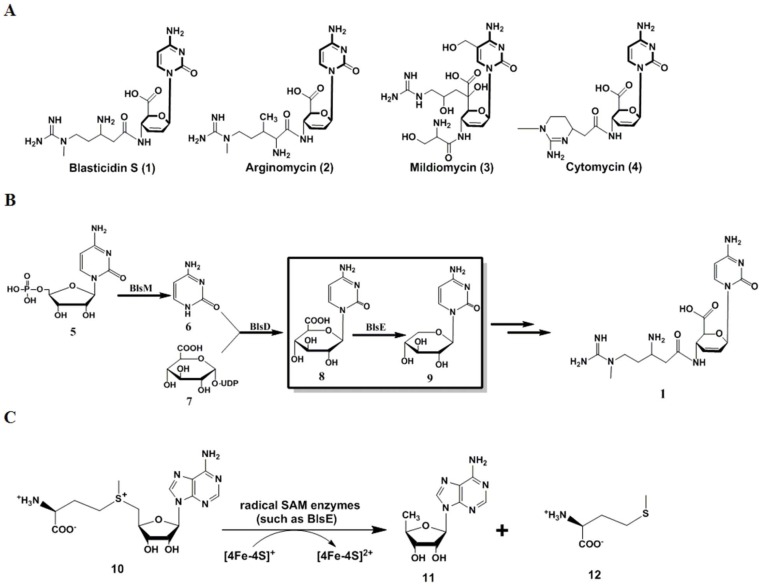
Radical SAM enzyme BlsE catalyzes decarboxylation in 1 biosynthesis. (A) Chemical structures of **1**–**4**. All compounds possess a cytosyl pyranoside core moiety (in bold lines). (B) Pathway for 1 formation *in vivo*, derived from cytidine monophosphate (**5**) and UDP-glucuronic acid (**7**). (C) General reaction scheme for radical SAM enzymes that causes SAM (**10**) cleavage into 5′-deoxyadenosine (**11**) and methionine (**12**).

Blasticidin S (**1**), a representative peptidyl nucleoside antibiotic produced by *Streptomyces griseochromogenes*, exhibits strong inhibitory activity against rice blast caused by *Pyricularia oryzae* Cavara in most regions of Asia and has replaced mercury fungicides [1]. It specifically inhibits protein synthesis in both prokaryotes and eukaryotes through inhibition of peptide-bond formation in the ribosomal machinery [2]. Compound **1** and its resistance gene (*bsr*) have been widely used for transgenic selection in eukaryotic cells [3], [4], [5], [6]. Unlike typical nucleosides, such as puromycin, nikkomycin, and pacidamycin, **1** features a pyranoside core moiety that is shared by arginomycin (2), mildiomycin (3), and cytomycin (4) (Figure 1A). This moiety is derived from cytosylglucuronic acid (CGA, **8**) which is formed by coupling UDP-glucuronic acid with free cytosine by CGA synthase BlsD/MilC (Accession number AAP03118/AFD20743) (Figure 1B). CGA and hydroxymethyl-CGA were accumulated upon mutation of *blsE* and *milG* in **1** and **3** biosynthetic pathway, respectively [7], [8]. Recently, ArgF (Accession number AGG35696), a homolog of BlsE/MilG (Accession number AY196214/AFD20747), was identified from compound **2** producer *Streptomyces arginensis* (NRRL 15941). The three protein homologs were predicted to be radical *S*-adenosyl-L-methionine (SAM, **10**) proteins with a typical motif C×××C×ФC (× represents any amino acid residue but cysteine, Ф usually refers to an aromatic amino acid). The biosynthetic gene clusters for **1** and **3** have been reported to share seven pairs of homologs [7], [9]. Each pair of homologous proteins in the pathways may govern the formation of a moiety with same or similar structure. Among the biosynthetic reactions, mechanism for the biochemical step catalyzed by BlsE/MilG remains obscure and most intriguing.

**Figure 2:**
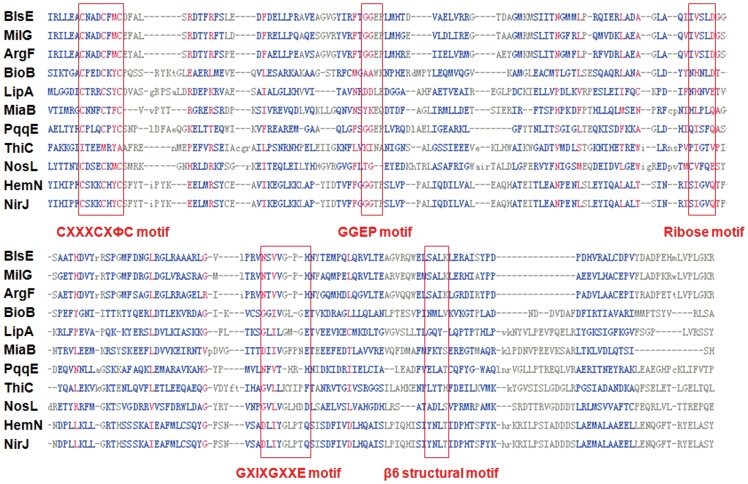
Sequence alignment of BlsE-like radical SAM homologs. Conserved residues within several structural motifs (red box) for typical radical SAM proteins are noted. The motifs include: C×××C×ФC motif, GGEP motif, ribose motif, G×I×G××E motif and β6 structural motif. Amino acid sequences were obtained from GenBank, including (1) BlsE from *Streptomyces griseochromogenes*; (2) MilG from *Streptomyces rimofaciens* ZJU5119; (3) ArgF from *Streptomyces arginensis* NRRL15941; (4) BioB from *Staphylococcus aureus* subsp. aureus ST398; (5) LipA from *Chlamydia trachomatis* IU824; (6) MiaB from *Thalassolituus oleivorans* MIL-1; (7) PqqE from *Pseudomonas poae* RE*1-1-14; (8) ThiC from *Acinetobacter baumannii* D1279779; (9) NosL from *Streptomyces. acutosus*; (10) HemN from *Hydrogenobaculum* sp. SHO; (11) NirJ from *Clostridium saccharoperbutylacetonicum* N1-4 (HMT).

The radical SAM protein superfamily currently contains thousands of members that participate in versatile biochemical reactions in the biosynthesis of coenzymes, vitamins, and antibiotics [10], [11], [12], [13]. Members of this family possess a common C×××C×ФC motif to coordinate with the [4Fe–4S] cluster. The common mechanism for the reaction catalyzed by a radical SAM protein is to generate 5′-deoxyadenosine (5′-AdoH, **11**) radical and methionine (**12**) from SAM, while **11** in turn serves as an oxidant to initiate radical transformation by abstracting a hydrogen radical from its substrate (Figure 1C). The following catalytic steps, specific to the given radical SAM enzyme, can lead to various outcomes, such as sulfur insertion (BioB [14], [15] in biotin synthase and LipA [16] in lipoyl synthase), methylthiolation (MiaB [17] in rRNA methylthiolation), and complex rearrangements (PqqE [18], [19] in pyrroloquinoline quinone biosynthesis, ThiC [20] in thiamine biosynthesis and NosL [21] in nosiheptide biosynthesis). Upon dithionite reduction, the iron–sulfur cluster can be reduced to [4Fe–4S]+, which is present in all active form of radical SAM proteins [22]. Besides the common C×××C×ФC motif, BlsE/MilG also contain the GGEP, ribose, G×I×G××E and β6 structural motifs that were demonstrated to coordinate with different moieties of SAM (i.e., adenine, ribose and methionine) in the studies of other radical SAM proteins (Figure 2) [23]. We previously observed that *in vivo* disruption of *milG* led to accumulation of hydroxymethyl-CGA [8]. CGA was therefore proposed as the direct substrate for the radical SAM protein BlsE in **1** biosynthesis. Herein, we report a full account of the characterization of BlsE and its catalyzed conversion of CGA to cytosylarabinopyranose (CAP, **9**).

## Results

### Expression and purification of BlsE

**Figure 3:**
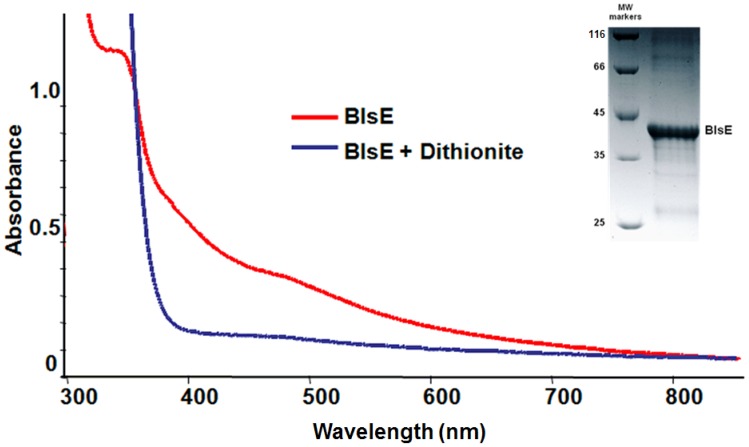
UV-vis spectrum of BlsE. The purified BlsE has a size of 40.2 KD as estimated by SDS-PAGE analysis; the red trace represents purified and reconstituted BlsE (50 *μ*M), whereas the blue trace represents BlsE reduced by 1 mM sodium dithionite.

Since the radical SAM protein has nucleated iron-sulfur cluster(s) that is highly susceptible to oxidation in the air, N-terminal His_6_-tagged BlsE was purified anaerobically using Ni-NTA column followed by concentration using Centricon YM-10 membrane (Millipore). A molecular mass of 38 kDa for BlsE plus the His_6_-tag predicts a purified protein of 40.2 kDa, consistent with the estimated size determined by gel filtration chromatograph (Figure 3, inset).

### Ultraviolet–visible (UV-vis) spectroscopic analysis of the reduced [4Fe–4S] cluster

The catalytic form of iron-sulfur cluster [4Fe–4S]^+^ was generated by using chemical reductant sodium dithionite in the anaerobic condition. The reduction of [4Fe–4S]^2+^ to [4Fe–4S]^+^ state in the process of the reaction was monitored by UV**-**vis spectroscopy. The decreasing shoulder at 420 nm and a remarkable shift at 360 nm indicate the presence of the [4Fe–4S]^+^ cluster (Figure 3) as reported for other radical SAM enzymes, such as BtrN [24] in butirosin biosynthesis, ThiC [25] in thiamin pyrimidine biosynthesis and NosL [21] in nosiheptide biosynthesis.

### Iron and sulfur content

The iron content was determined by measuring the maximum absorbance wavelength of Fe^2+^-ferene complex at 593 nm. The resulting data showed that unreconstituted His_6_-tagged BlsE contained approximately 1.4±0.5 iron equivalents per monomer of protein. In contrast, the fully reconstituted His_6_-tagged BlsE contained approximately 6.8±0.4 iron equivalents per monomer, which clearly indicated iron increased by nearly 5 times during reconstitution process. It was shown that the unreconstituted His_6_-tagged BlsE contained approximately 1.6±0.3 sulfur equivalents per monomer of protein, while the fully reconstituted His_6_-tagged BlsE contained nearly 8.5±0.4 sulfur equivalents per monomer. These data suggested that the BlsE monomer may contain two [4Fe-4S] clusters after *in vitro* chemical reconstitution.

### EPR spectroscopy of the [4Fe–4S] cluster in BlsE

**Figure 4:**
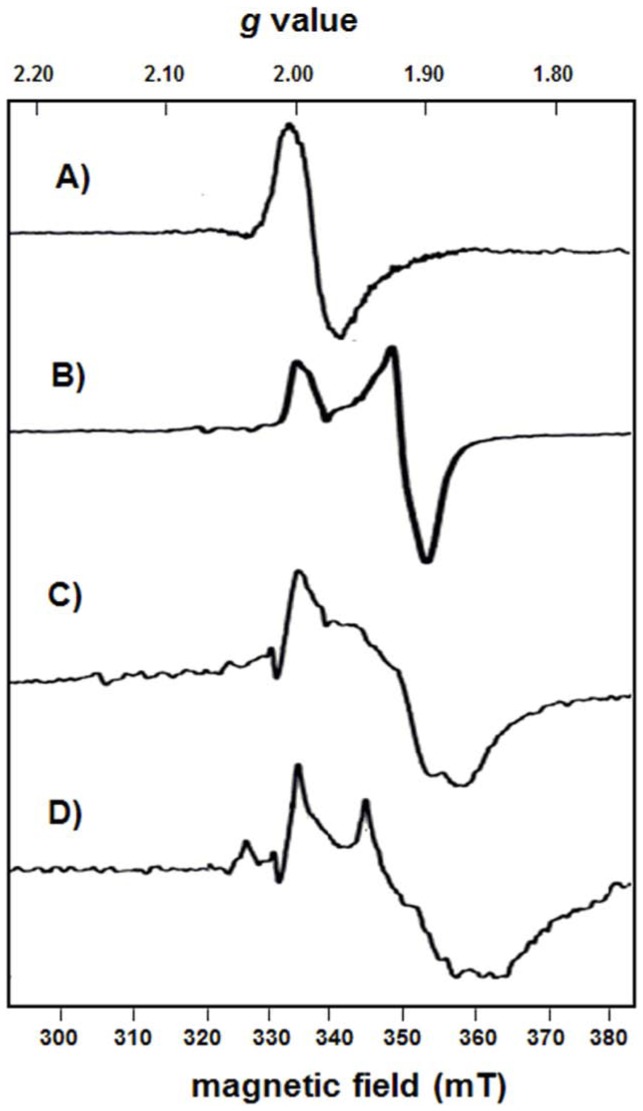
EPR spectra of BlsE under various conditions. (A) As-isolated sample of BlsE (120 *μ*M). (B) Reconstituted BlsE reduced by dithionite before loading into an EPR tube anaerobically. (C) B with 1 mM SAM. (D) C with 1 mM CGA. EPR conditions used were as follows: microwave frequency, 9.390 GHz; microwave power, 10 *μ*W; modulation amplitude, 2 Gauss; modulation frequency, 100 kHz; temperature, 13K. Each spectrum is the average of 80 scans.

Electron paramagnetic resonance (EPR) spectroscopy has been an essential tool for gaining full understanding of the radical SAM enzymes [26]. In particular, EPR spectroscopy has played a key role in identifying iron-sulfur clusters and, together with Mössbauer spectroscopy, in characterizing cluster types including its oxidation states and the catalytically active cluster in radical SAM enzymes [27]. From our results, the *g* factor (the value of the dimensionless magnetic moment) obtained was 2.01 for as-isolated BlsE (Figure 4A), characteristic of a [3Fe–4S]^+^ cluster (spin state (S) 1/2), which was recognized as the product of air oxidation of the [4Fe–4S]^3+^ cluster possessed by the inactive enzyme. Similar *g* factor values were reported for several radical SAM enzymes, such as the anaerobic ribonucleotide reductase with a *g* factor centered at 2.017 [28], and D-desosamine oxidative dehydrogenase (DesII) with a *g* factor centered at 2.01 [29]. Furthermore, this result was supported with the iron and sulfur content analysis which generated Fe^2+^/S^2−^ ratios averaging about 0.8, higher than expected for a [3Fe–4S] cluster. The discrepancies between the spectrum of as-isolated BlsE and that of the synthetic [3Fe-4S] cluster most likely resulted from a mixture of small amount of [4Fe-3S]^3+^ cluster existed in BlsE. However, as the [4Fe–4S]^2+^ cluster usually exhibit silence in EPR spectra, it seems that the [4Fe–4S] cluster of BlsE was unstable in the absence of SAM and could easily lose one iron to assume the [3Fe–4S] form [30]. In contrast, when reduced by sodium dithionite, the reconstituted protein showed two peaks with *g* factors of 2.02 and 1.93 (Figure 4B), which suggested the generation of a [4Fe–4S]^+^ cluster as reported to be possessed by typical radical SAM proteins [24]. After adding **10** to the reaction mixture, a rhombic spectrum with *g* values of 2.00, 1.93 and 1.86 was observed (*g*_av_  = 1.93) (Figure 4C), similar to that of the [4Fe–4S]^+^ cluster in DesII with *g* values of 2.01, 1.96, and 1.87 (*g*_av_  = 1.95) [29]. Adding both **8** and **10** to the reaction led to a significant EPR spectral change, indicating that a substrate radical was generated in the EPR spectrum through the coordination of BlsE nucleophilic groups with the active iron of the [4Fe–4S] cluster (Figure 4D). The significant EPR signal caused by this unstable conformation indicated the strong interaction between substrate **8** and [4Fe–4S] cluster. Samples were also analyzed in the absence of glycerol, and the results showed that glycerol had little effect on the EPR spectra of BlsE (Figure S1). These results confirmed that the EPR spectral changes were caused by the interaction between **8** and the [4Fe–4S] cluster, not glycerol, in the reaction mixture.

### Activity assay of the BlsE-catalyzed reactions

**Figure 5:**
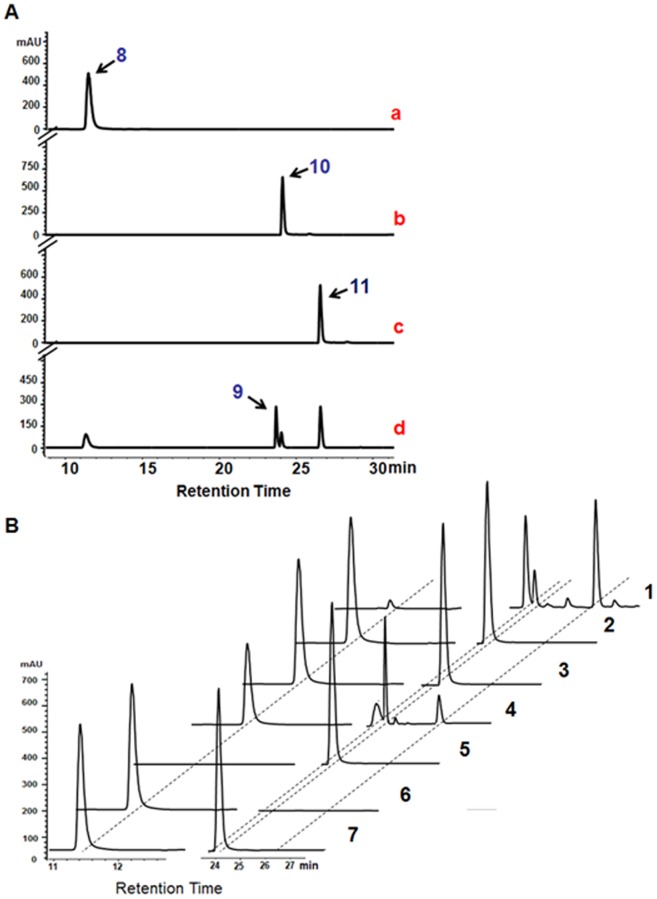
Activity of iron–sulfur cluster-reconstituted purified BlsE. (A) HPLC traces of BlsE catalytic reactions with **8**: (a) standard **8**; (b) standard **10**; (c) standard **11**; (d) **8**+**10**+BlsE. (B) Composite HPLC traces of various treatment. Sample 1 represents full reaction (CGA, SAM, dithiothreitol, sodium dithionite, and BlsE). Samples 2 and 3 represent *in vitro* BlsE reaction incubated aerobically and BlsE purified under aerobic conditions, respectively. Samples 4–7 represent full reactions without dithiothreitol, **8**, **10**, or sodium dithionite, respectively. All the experiments used sodium dithionite as the reductant.

The substrate CGA was chemically synthesized starting from commercial N-acetylcytosine via two-step synthesis (Figure S2) and the structure was confirmed with ^1^H NMR spectra (Figure S3–S4). The complete reaction of fully reconstituted BlsE with CGA yielded two new peaks in the chromatograms during HPLC analysis that corresponded to compounds **9** and **11** (Figure 5A, d), respectively. However, when BlsE was purified aerobically or the incubation was operated in the air, no new peaks corresponding to the desired products were observed (Figure 5B, Samples 2, 3 respectively), which indicated that this enzyme must work under strict anaerobic conditions. Additionally, BlsE reactions without **8**, **10**, or sodium dithionite revealed that no products were detected in the reaction mixtures (Figure 5B, Samples 5, 6 and 7, respectively). Moreover, since dithiothreitol can considerably increase the rate of the cleavage reaction of the SAM [31], its absence in our reaction system led to a significant reduction in the formation of **9** (Figure 5B, Sample 4). The same peak fragments were combined, concentrated and analyzed by NMR (^1^H NMR (500 MHz, *methanol-d4*) and ^13^C NMR (125 MHz, *methanol-d4*) spectra. The signals of **9** are as follows: δ_H_ 7.70 (d, 2H), 6.06 (d, 1H), 5.55 (d, 1H), 3.97 (m, 1H), 3.65 (dd, 1H), 3.56 (dd, 1H), 3.41 (d, 1H) and 3.32 (d, 2H) and δ_c_ 167.69, 159.23, 143.50, 97.52, 86.03, 79.25, 73.25, and 71.25 (Figure S5-S6). Furthermore, by using comprehensive 2D NMR techniques including ^1^H-^1^H COSY, HSQC and HMBC, the structure of **9** was determined (Figure S7-S9). The High Resolution Electrospray Ionization Mass Spectrometer (HR-ESI-MS) data are listed as follows: for isolated **9,** HR-ESI-MS *m/z* calcd 244.0855, found 244.0853 (Figure S10); for isolated **11,** HR-ESI-MS *m/z* calcd 252.1018, found 252.1152) (Figure S11).

### Time dependence of cytosylarabinopyranose and 5′-AdoH formation during *in vitro* BlsE reaction

**Figure 6:**
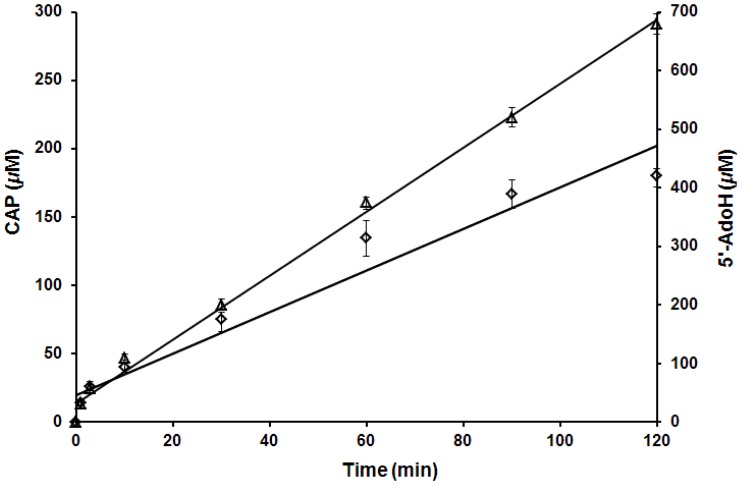
Time dependence of CAP and 5′-AdoH production. Time dependence of CAP (◊) and 5′-AdoH (Δ) formation in the reaction with CGA. Error bars show SD, each data point represents the average of three replicates.

To understand the relationship between the reductive cleavage of SAM and oxidative decarboxylation catalyzed by BlsE, we measured the time-dependent formation of cytosylarabinopyranose (CAP, **9**) and consumption of SAM (**10**), indicated by the formation of 5′-AdoH (**11**). In the first 5 minutes, equal molar of **11** and **9** were formed during the BlsE-catalyzed reaction (Figure 6). This clearly proved that in the initial stage of the reaction one molecule of 5′-deoxyadenosine radical (5′-Ado•, **13**), which is generated from reductive homolytical cleavage of **10** by BlsE, can capture one electron from CGA to form **11** and catalyze decarboxylation of CGA to form **9**. The first order rate constants of the BlsE reaction with CGA was 1.93 min^−1^, which allowed the BlsE-catalyzed reaction to be used for quantitative analysis of **9** formation. However, with the increasing formation of **11**, the generation rate of **9** obviously lagged after 60 min compared to formation of **11**, suggesting that excess **11** inhibits **13** from capturing electron from **8**, and thus leading to reduced rate of **9** formation.

### Activity assay of BlsE-catalyzed reactions by flavodoxin (Fld) and flavodoxin reductase (Fpr)

**Figure 7:**
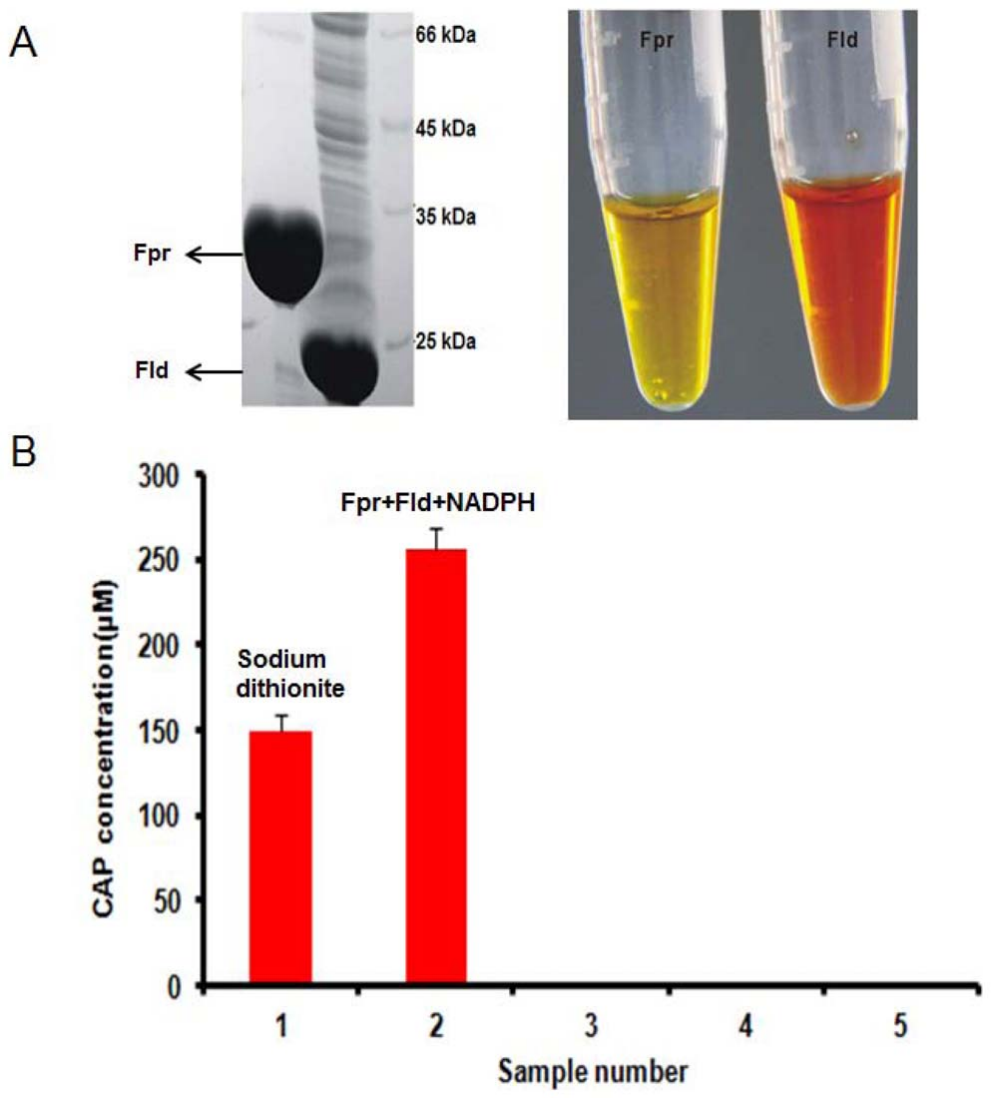
Activity assay of BlsE-catalyzed reaction by Fld, Fpr and NADPH. (A) Gel electrophoresis analysis (SDS-PAGE) of Fpr and Fld on the left side. Fpr and Fld came out as golden and light brown in 50 mM HEPES (pH 8.0) on the right side, respectively. (B) Activity of the natural reduction system. Sample 1 represents the reaction mixture containing SAM, CGA, sodium dithionite, and BlsE. Sample 2 has same reaction mixture as sample 1 but with sodium dithionite substituted by NADPH, Fpr and Fld. Samples 3–5 represent reaction 2 without NADPH, Fpr and Fld, respectively. Error bars show SD, each data point represents the average of three replicates.

Although chemical reductants such as 5-deazaflavin and dithionite are commonly used in the process for the reduction of [4Fe–4S] cluster, Fld, Fpr and NADPH have also been demonstrated as effective reducing systems for several radical SAM enzymes. In this system, the electron from [4Fe–4S]^2+^ was transferred to [4Fe–4S]^+^ stepwise from NADPH to NADP, following by the recycle of Fld and Fpr in the *in vivo* reducing system. Fld and Fpr genes were cloned from *E. coli* BL21 (DE3). The expressed Fld and Fpr proteins were purified and analyzed by SDS-PAGE. Purified fractions revealed two bands of ca. 22 kDa and 30 kDa in size, consistent with calculated molecular weight of Fld and Fpr, respectively (Figure 7A). Additionally, Fpr and Fld solutions had golden and brown color, respectively (Figure 7A), suggesting the presence of a flavin mononucleotide cofactor and a flavin adenine dinucleotide in the active site, respectively. The results indicated that **9** could also be generated when using NADPH, Fld and Fpr reducing system and the amount of **9** was 1.5 times higher than that when sodium dithionite was used (Figure 7B).

### Mutagenesis study of the conserved motif of BlsE

**Figure 8:**
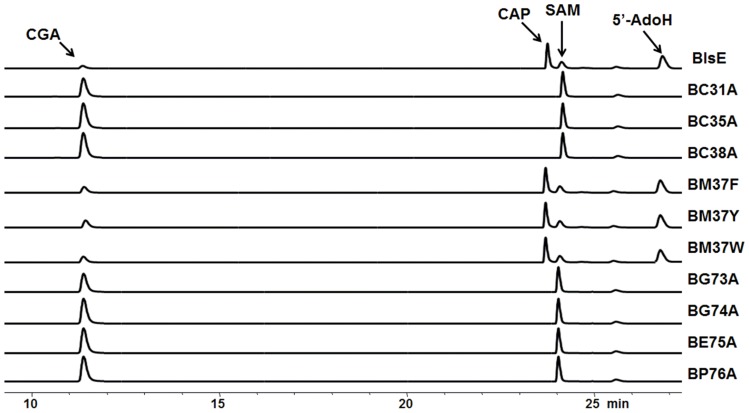
*In vitro* assays of the mutants. The BlsEC31A, C35A and C38A (abbreviated as BC31A, BC35A and BC38A in figure) represent respective mutant proteins in which cysteine in the C×××C×MC motif of BlsE was replaced by alanine; BM37F, BM37Y and BM37W (similarly abbreviated) represent mutant proteins in which methionine in the same motif was replaced by phenylalanine, tyrosine or tryptophan, respectively. BG73A, BG74A, BE75A and BP76A represent mutant versions of BlsE in which each amino acid in the GGEP motif was changed into an alanine.

Multiple alignment of BlsE with its homologs revealed a C_31_×××C_35_×M_37_C_38_ motif and a G_73_G_74_E_75_P_76_ motif (Figure 2). The first one was crucial for binding the [4Fe–4S] cluster for the coordination with SAM; the latter one was required for binding with the amino group of methionine moiety of SAM, thus ensuring proper ligation of the cluster's unique iron and correct orientation of the SAM methionyl moiety [32]. We constructed a series of mutants for the replacement of cysteine residues of C_31_×××C_35_×M_37_C_38_ with alanine, and Met_37_ with aromatic amino acids, phenylalanine, tyrosine or tryptophan, individually. Similarly, we replaced each amino acid residue of the G_73_G_74_E_75_P_76_ motif with alanine. The mutant proteins were purified and analyzed with SDS-PAGE (Figure S12). The results showed that Cys is required for BlsE activity because all Cys-replaced mutants could not convert **8** to **9** (Figure 8). In other radical SAM proteins, Met_37_ is usually an aromatic acid, typically phenylalanine or tyrosine that interacts with the adenine of SAM. However, in our system, minimal effects were observed on **9** production in the BM37F, BM37Y and BM37W mutants, indicating that this site was not vital for BlsE catalytic activity (Figure 8). On the other hand, none of the GGEP motif mutants showed any activity for BlsE-catalyzed reactions, demonstrating that binding via the GGEP motif with methionine moiety of SAM was essential for BlsE activity. The failure of **9** formation in the reactions by mutants BG73A and BG74A indicated that the methyl group of alanine may block the steric conformation of binding site of [4Fe–4S] cluster. Nevertheless, the identity of GGE residues has not always been conserved. For example, the residues in BioB are AAW instead of GGE, while the structure and function of this motif was equivalent to a classic GGE motif [33]. Similarly, lack of production of **9** could be observed for mutants BE75A and BP76A. This provided the evidence that substitutions of glutamic acid or proline into alanine may influence the SAM binding pocket with [4Fe–4S] cluster, in other words, the hydrogen bond formation between the active iron and amino group or carboxyl group of methionyl moiety of SAM was essential for BlsE activity. Further study of 3D crystal structure of BlsE is in progress.

### Determination of the kinetic parameters for reactions catalyzed by BlsE and select mutant proteins

A 500-*µ*L reaction volume was used for reactions containing lower substrate concentrations to facilitate HPLC analysis. Enzyme reactions were performed with varying concentrations of substrate **8** (0.05–800 *µ*M) in the presence of a constant concentration of **10** (1 mM). The results were fitted to the Michaelis–Menten equation by nonlinear regression and yielded a *k*_cat_ of 1.62±0.3 min^−1^ and a *K*_m_ of 1.93±2.4 *µ*M for **8** (for BlsE), while the mutants BM37F, BM37Y and BM37W have *k*_cat_ values of 1.46±0.3 min^−1^, 1.24±0.5 min^−1^ and 1.43±0.3 min^−1^, respectively, and *K*_m_ values of 2.15±3.8 *µ*M, 1.96±4.2 *µ*M, and 2.17±3.8 *µ*M for **8**, respectively (Table 1). The results indicated that BlsE has a higher binding affinity for with CGA than the mutants since the *k*_cat_/*K*_m_ for BlsE was 0.84 *μ*M^−1^min^−1^, much higher than those of BM37F, BM37Y and BM37W proteins, which have the *k*_cat_/*K*_m_ ratios of 0.68, 0.63, and 0.70 *μ*M^−1^min^−1^ respectively (Table 1). Additionally, such results could provide guidance for us while we analyze the crystal structure of BlsE, MilG, ArgF, NosL and NocL, which all share a methionine in place of an aromatic amino acid, commonly found at this position in other members of this enzyme family.

**Table 1:** Kinetic parameters for BlsE and select mutant versions of BlsE.

Enzyme*	*k*cat (min^−1^)	*K*m (*μ*M)	*k*cat/*K*m (*μ*M^−1^min^−1^)
BlsE	1.62	1.93	0.84
BM37F	1.46	2.15	0.68
BM37Y	1.24	1.96	0.63
BM37W	1.43	2.04	0.70

* Enzyme kinetic parameters were tested using CGA as the substrate with the concentration ranged from 0.025–800 *μ*M, as indicated. All the reactions were incubated at 25°C.

## Discussion

BlsE was predicted as a radical SAM enzyme as it has five conserved motifs that serve to coordinate with the iron-sulfur cluster and SAM, characteristic of this family of proteins. To gain insights into the reaction mechanism, we first investigated [4Fe–4S] cluster of BlsE by UV-vis absorption and the EPR spectra. As expected, BlsE contains the [4Fe–4S] cluster that is characteristic of the radical SAM protein. The content of iron and sulfur indicated that BlsE may contain two [4Fe–4S] clusters, which is supported by the fact that removal of two electrons is theoretically necessary for the oxidative decarboxylation of CGA to obtain CAP. We assume that the C_31_×××C_35_×M_37_C_38_ motif binds one of the [4Fe–4S] clusters which coordinates binding with SAM, while another motif (C_317_×C_319_××C_322_) may provide the binding site for the second iron-sulfur that is supposed to coordinate with the nucleophilic groups of CGA. A good example is BtrN, which contains two [4Fe–4S] clusters to coordinate with SAM and 2-deoxy-*scyllo*-inosamine (DOIA), respectively [34]. This assumption was partly supported by the generation of a significant EPR signal when SAM and CGA were added into the reaction mixture (Figure 4D), suggesting that one active iron of the [4Fe–4S] cluster in BlsE coordinates with the nucleophilic groups of CGA. Similar EPR signals were also found for several other radical SAM enzymes, such as the pyruvate formate-lyase-activating enzymes [27], in which the EPR signal was generated due to the alteration in [4Fe–4S] cluster geometric conformation. In addition, glycerol is known to be able to coordinate with the [4Fe–4S]^+^ cluster, resulting in spectroscopic changes similar to those observed for SAM coordination [35]. In our study, the EPR spectrum of BlsE in the absence of glycerol was nearly identical to that containing glycerol, suggesting that glycerol does not coordinate with [4Fe–4S]^ +^ cluster in the presence of SAM. This evidence helped us eliminate the effect of glycerol on the reactions. In addition, small changes were observed in the reduced BlsE with SAM, because the *g* = 2.00 feature was more pronounced when glycerol was used alone in the mixture. These findings are similar to those reported for NocL, which is involved in nocathiacin I biosynthesis, in which the EPR signal also changed when L-Trp was added to the reaction mixture [36].

Since dithionite was not the physiologically reductant, assays were performed to test whether flavodoxin and flavodoxin reductase could also support this reaction. Flavodoxin is a flavin mononucleotide-containing protein that provide reducing equivalents in a variety of enzyme-catalyzed reactions [37]. In reactions using the reconstituted functional BlsE, flavodoxin, flavodoxin reductase and NADPH were demonstrated to be more effective reducing system than sodium dithionite. In this system, the electron from [4Fe–4S]^2+^ to [4Fe–4S]^+^ was transferred stepwise from NADPH to NADP, followed by the cycle of flavodoxin and flavodoxin reductase in the *in vivo* reducing system. This *in vivo* reducing system was also employed in studies of NosL- and DesII-catalyzed reactions instead of chemical reductant sodium dithionite [21], [29].

To determine the functional involvement of some key amino acids of BlsE in decarboxylation, we systematically generated mutations which changed conserved amino acid residues in the C×××C×ФC and GGEP motifs and then measured the yields of CAP. Our site-directed mutagenesis studies of the conserved cysteines in the C×××C×ФC motif showed results consistent with previous reports involving other radical SAM enzymes [36], confirming BlsE as a typical radical SAM enzyme. Surprisingly, our enzyme contains a methionine residue at the Ф position of the motif, usually reserved for aromatic acid, in other radical SAM proteins [19], [38]. It was suggested that an aromatic amino acid at this position would be important for hydrophobic interactions with the adenine moiety of SAM [39]. We found that mutant versions of BlsE with aromatic acids at this position had slightly lower activity for the formation of CAP, suggesting this site is not vital for BlsE activity. This finding may help us understand the diverse reactivity of this amino acid position and should be useful for the 3D crystal structure of this family of enzymes including MilG, ArgF, NosL and NocL (Figure 2). Interestingly, the *in vitro* assay results of GGEP motif mutants were slightly different from other radical SAM enzymes. For example, in BioB, the GGE residues were replaced by AAW, but the enzyme still maintains its activity [33]. In our system, BG73A and BG74A substitutions abolished BlsE activity, possibly because the extra side group (methyl group) narrowed the barrel for active site or blocked the interaction between the residue and amino group of SAM. Similarly, BE75A and BP76A substitutions eliminated the BlsE activity, suggesting that these two residues were crucial for the interaction of methionyl moiety and the active iron of [4Fe–4S] cluster [32]. Further crystallography study of this enzyme is in progress.

**Figure 9:**
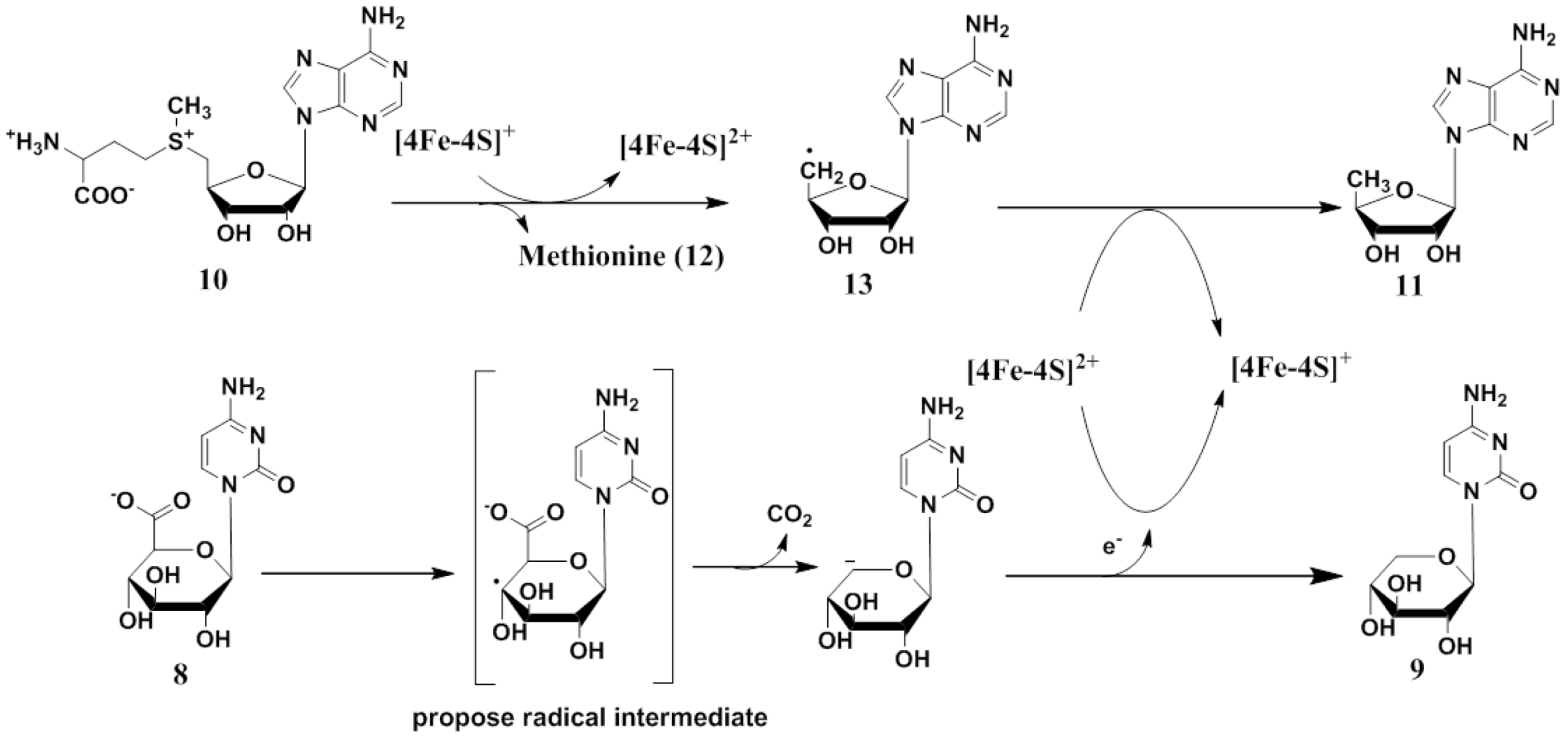
Proposed mechanism of BlsE-catalyzed reaction. BlsE catalyzes the decarboxylation at C5 position of **8**, leading to the formation of **9**. SAM (**10**) serves as the source of methionine (**12**) and 5′-Ado radical (**13**), the latter could abstract the hydrogen from **8** and generate 5′-AdoH (**11**).

The proposed catalytic mechanism of BlsE reaction is shown in Figure 9. Briefly, 5′-deoxyadenosine radical was first generated by reductive cleavage of SAM with the [4Fe–4S]^+^ cluster using sodium dithionite as reductant, followed by abstracting a hydrogen atom from the substrate CGA to yield an unstable substrate radical. This likely carboxyl radical which has been observed by EPR may undergo decarboxylation to give rise to carbon dioxide and the possible carbanion intermediate. Further, 5′-AdoH was generated as the intermediate by transferring one electron to the [4Fe–4S]^2+^ cluster, resulting in [4Fe–4S]^+^ cluster regeneration and CAP formation. Thus, the radical intermediate-reduced [4Fe–4S]^2+^ may cause a conformational change which further raised the reduction potential of [4Fe–4S] cluster, while the electron from substrate radical intermediate is probably transmitted to [4Fe–4S]^2+^ cluster, which accepts the electron to give itself to the reduced form.

Coproporphyrinogen III oxidase (HemN), another member of this enzyme family catalyzing a oxidative decarboxylation reaction, converts coproporphyrinogen III to protoporphyrinogen in chlorophyll biosynthesis, and is the only radical SAM enzyme found to catalyze a two-step oxidative decarboxylation using one [4Fe–4S] center binding to two SAM cofactors [40]. However, in this case, an uncharacterized electron acceptor in a cell-free extract of *E. coli* was required to complete the reaction. Surprisingly, in our work, it appears that the electron from CAP can be transmitted to an electron acceptor, while the [4Fe–4S]^2+^ also could be reduced to the [4Fe–4S]^+^ for the next cycle. As mentioned above, the electron released from the substrate radical intermediate is somehow transferred to the [4Fe–4S]^2+^ cluster to produce CAP and regenerate the [4Fe–4S]^+^ cluster for the next catalytic cycle. Another example of radical SAM enzyme is NirJ which is involved in tetrapyrrole biosynthesis; it catalyzes the decarboxylation of siroheme to yield didecarboxysiroheme, which is then converted to Fe-coproporphyrin by oxidative decarboxylation of two acetyl side chains [41], [42]. Given the high similarity between BlsE and NirJ (35% amino acid identity and 50% similarity), it is reasonable to propose that BlsE may also catalyze similar reactions.

In summary, we have successfully reconstituted the BlsE, which catalyzed carbon chain cleavage of C5 glucoside in CGA to yield CAP. Also, we have demonstrated via EPR and UV-vis spectroscopy that this enzyme requires a reduced [4Fe–4S] cluster for catalysis. To our knowledge, this is the first report detailing the *in vitro* decarboxylation of a glucoside moiety by a member of the radical SAM superfamily, which expands the functional diversity of this enzyme family. Further characterization of this unique mechanism is ongoing in our laboratory. Elucidating unique functions of BlsE and other radical SAM enzymes may lead to the discovery of novel metabolic pathways and facilitate chemical and biochemical applications of peptidyl nucleoside antibiotics.

## Materials and Methods

### General

SAM was purchased from Sigma-Aldrich Corporation. Dithiothreitol and ferrous ammonium sulfate were obtained from Sangon Biotech Co. Ltd. Cysteine, sodium dithionite and N-acetylcytosine were purchased from TCI Inc. All media were purchased from Oxoid Ltd. HPLC-MS analysis was operated using an Agilent 1100 Series System with TC-C18 (5 *μ*m, 4.6×250 mm) equipped with a diode array UV**-**vis detector and an in-line fluorescence detector. ^1^H and ^13^C spectra were recorded using Agilent instruments (^1^H frequencies  = 500 MHz; corresponding ^13^C frequencies  = 125 MHz). Chemical shifts were expressed as parts per million relative to TMS. Primers used are shown in Table S1.

### Cloning, expression and purification of BlsE, flavodoxin (Fld) and flavodoxin reductase (Fpr)

For BlsE overexpression, the *blsE* gene was amplified from the total DNA of *Streptomyces griseochromogenes* via PCR and the 1.1-kb PCR product was cloned to pBluescript SK+. After digestion with the restriction enzymes *Nde*I and *Eco*RI, the resulting insert fragment was cloned to the same enzyme sites of pET-28a. The insert of the recombinant plasmid was sequenced and confirmed, and the correct plasmids were then transformed into *E. coli* BL21 (DE3).

For flavodoxin (Fld, accession number was YP_002998478) and Fld reductase (Fpr, accession number was YP_003001491) expression plasmid construction, the PCR products containing *fld* and *fpr* genes (derived from *E.coli* BL21) were cloned similarly into pBluescript SK+ as intermediate constructs, from which the *fld* and *fpr* gene fragments were released from vector by *Eco*RI and *Nde*I and inserted into the same sites of pET-28a to generate recombinant plasmids, respectively.

All strains were grown from overnight cultures in fresh Luria–Bertani medium with 50 *μ*g mL^−1^ kanamycin at 37°C. When the optical density reached 0.6–0.8 at 600 nm, the protein over-expression was induced with 0.2 mM IPTG and continuously grown at 16°C for another 20–24 h. After harvesting by centrifugation at 6,000 rpm for 10 min, the cells were stored at −80°C until use. For BlsE purification, all processes were carried out in an anaerobic box (Coy Laboratory Product Inc., USA) under a strictly controlled oxygen concentration below 2 ppm. The proteins were resuspended in an ice-cold lysis buffer (50 mM HEPES, pH 8.0). After being lysed by a sonicator (Sonics Materials, Inc., USA), the cell debris was removed by centrifugation at 15,000 rpm for 60 min, and the supernatant was incubated with 5 mL of Ni–NTA resin (Invitrogen) pre-equilibrated with lysis buffer. The resins were loaded onto a polypropylene column and washed with lysis buffer until there was no UV absorption at 280 nm. After additional wash with 5 column volumes of wash buffer (50 mM HEPES, 300 mM NaCl, and 50 mM imidazole, pH 8.0), His-tagged proteins were eluted from the column with elution buffer (50 mM HEPES, 300 mM NaCl, and 500 mM imidazole, pH 8.0). The resulting proteins were concentrated with ultracentrifugation filter YM-10 membrane (Millipore), their purity was confirmed by 12% SDS-PAGE analysis, and their concentrations were determined by Bradford assay using bovine serum albumin as standard.

### Iron–sulfur content determination

Iron content was measured by determining the optical density of the Fe^2+^–ferene complex at the wavelength of 593 nm [43]. Specifically, the protein solution was diluted with ddH_2_O to a final volume of 100 *µ*L. In total, 100 *µ*L of 1% HCl was added to the solution, and the mixture was incubated for 10 min at 80°C to denature the protein. Next, 500 *µ*L of ammonium acetate (7.5%, w/v), 100 *µ*L of ascorbic acid (4%, w/v), 100 *µ*L of SDS (2.5%, w/v), and 100 *µ*L of ferene (1.5%, w/v) were added, resulting in a blue solution. The mixture was vortexed and centrifuged at 9,000 rpm for 5 min. The optical density at 593 nm was measured using an Ultraspec 3000 photometer (Pharmacia Biotech). A standard curve was obtained by measuring iron standards with an iron range of 2–40 nmol.

For the measurement of sulfide content, a small amount of the protein solution was diluted with ddH_2_O to a final volume of 200 *µ*L. After the addition of 600 *µ*L of zinc acetate (1%, w/v) and 50 *µ*L of sodium hydroxide (7%, w/v), the solution was carefully mixed, incubated for 15 min at room temperature, and centrifuged at 3,000 rpm for 10 s. Subsequently, 150 *µ*L of DMPD (*N*, *N*-dimethyl-*p*-phenylene-diamine monohydrochloride) (0.1%, w/v, in 5 M HCl) and 150 *µ*L of FeCl_3_ (10 mM in 1 M HCl) were added. The released inorganic sulfide was first absorbed onto zinc to generate zinc sulfide and then released to react with DMPD to give methylene blue, which could be detected by the UV**-**vis spectrum at 670 nm after 20 min of incubation at room temperature [44]. Sulfide standards with a sulfide content range of 2–50 nmol were used to obtain a standard curve.

### *In vitro* anaerobic chemical reconstitution of the BlsE

The as-isolated protein was yellow brown. Dithiothreitol (5 mM) was added into the solution on ice for 15 min, followed by 1 mM ferrous ammonium sulfate for 10 min. Sodium sulfide was added to the same final concentration (1 mM) in a similar way. The mixture was allowed to incubate on ice for 30 min, after which 2.5 mL of reconstituted protein was loaded onto a 10 DG desalting column (Bio-Rad) pre-equilibrated with lysis buffer. After being eluted with 3 mL of lysis buffer, the dark brown protein was collected, sealed tightly, and stored at −80°C until use.

### UV-vis and EPR spectroscopy

For UV-vis assay, samples were prepared in a glove box in the presence of 5 mM dithiothreitol. The reconstituted BlsE was treated with sodium dithionite and the reactions were monitored by UV-vis spectra at a full wavelength scan of 300–900 nm. For EPR assay, samples were prepared including 10 mM dithiothreitol and 120 *μ*M BlsE in each EPR tube. BlsE (with or without glycerol) was reduced with excess sodium dithionite (10 mM) at 25°C for 30 min. SAM (1 mM) and CGA (500 *μ*M) were added to the reduced sample in the same manner into an EPR tube, frozen in liquid nitrogen, and stored on dry ice before recording the spectra. EPR spectra were recorded using a Bruker EMX Plus 10/12 spectrometer (Bruker Co., Ltd., Germany) equipped with an Oxford ESR910 liquid helium continuous flow cryostat (Oxford Instrument Co., Ltd., UK) at the National High Magnetic Field Laboratory of the Chinese Academy of Sciences, Hefei, China. Acquisition conditions for the Fe-S cluster analysis are: microwave frequency, 9.390 GHz; microwave power, 1 mW; field modulation amplitude, 5 Gauss; modulation frequency, 100 kHz; temperature, 13K. EPR spectra of the iron-sulfur clusters were simulated by SimFonia (Bruker, Co., Ltd., Germany).

### Activity assay of BlsE-catalyzed reactions

Assays were performed in a glove box under strictly anaerobic conditions with an oxygen concentration below 2 ppm. The assay reactions contained 1 mM SAM, 5 mM dithiothreitol, 1 mM sodium dithionite, 500 *μ*M CGA, and 15 *μ*M protein in 50 mM HEPES buffer (pH 8.0). Reactions were initiated with SAM and incubated at 25°C for 3 h, 5% (v/v) trifluoroacetic acid (TFA) was added to the reaction mixture, and the precipitates were removed by centrifugation. The supernatant was subjected to HPLC-MS analysis. The HPLC-MS experiments used a two-stage linear gradient: from 2% to 40% acetonitrile, in 20 mM ammonium acetate, pH 5.5, versus ddH_2_O over 30 min, followed by 40% to 2% acetonitrile in 20 mM ammonium acetate, pH 5.5, versus ddH_2_O over 20 min at a flow rate of 0.3 mL min^−1^. For the products isolated, an Agilent semi-prepared column (ZORBAX ODS 250×9.4mm, 5 *µ*m) was used. The mixture was subjected to separation by using the column coupled with the detector set at 260 nm and eluted with a 30-min linear gradient of 2% to 20% acetonitrile at a flow rate of 1 mL min^−1^.

### Quantitative analysis of product formation *in vitro*

Time course analysis including various intervals at 1, 3, 10, 30, 60, and 120 min was performed. At each interval, a 50-*μ*L reaction aliquot was collected and terminated by addition of 5% TFA. CAP and 5′-AdoH production was measured by HPLC. The standard curves were generated with the authentic 5′-AdoH and CAP so as to correlate peak area with the amount of 5′-AdoH and CAP.

### Activity assay of BlsE-catalyzed reactions using Fld, Fpr and NADPH as the reducing system

The natural reduction system assay contained 50 mM HEPES buffer (pH 8.0), 500 *μ*M CGA, 1 mM SAM, 1 mM NADPH, 25 *μ*M Fld, 20 *μ*M Fpr, and 15 *μ*M reconstituted BlsE in 200 *μ*L of volume. The mixture was incubated at 25°C for 1 h and stopped with 5% TFA. After centrifugation at 12,000 rpm for 5 min, the supernatant was subjected to HPLC assay.

### Mutagenesis study of the conserved motif in BlsE

Site-directed mutational analysis of BlsE was performed using a KOD-Plus-Mutagenesis Kit with the pET-28a-*blsE* plasmid as the template. After PCR with KOD-Plus DNA polymerase (TOYOBO), the obtained DNA fragments were digested for 1 h with *Dpn*I to remove the methylated template plasmid. Further steps were carried out according to the manufacturer’s instructions. The plasmids containing the confirmed desired mutations (pET-28a-BC31A, pET-28a-BC35A, pET-28a-BM37F, pET-28a-BM37Y, pET-28a-BM37W, pET-28a-BC38A, pET-28a-BG73A, pET-28a-BG74A, pET-28a-BE75A and pET-28a-BP76A) were used to transform into *E. coli* BL21 (DE3) cells for subsequent expression. The resulting plasmids were introduced into *E.coli* BL21 (DE3) to yield the recombinant strains BC31A (A×××C×ФC), BC35A (C×××A×ФC), BC38A (C×××C×ФA), BG73A (AGEP), BG74A (GAEP), BE75A (GGAP), and BP76A (GGEA). As the Ф amino acid in the C×××C×ФC motif for most radical SAM proteins belongs to an aromatic amino acid [45], Met_37_ was thus changed into phenylalanine, tyrosine or tryptophan to yield BM37F (C×××C×FC), BM37Y (C×××C×YC) and BM37W (C×××C×WC) to determine the effects of amino acid substitutions on enzyme activity.

### Determination of the kinetic parameters for BlsE-catalyzed reaction

The steady-state kinetic parameters of the BlsE-catalyzed reactions were determined by activity assay as previously described above. Specifically, 10 *µ*M enzyme (either BlsE, the mutant versions BM37F, BM37Y or BM37W), 1 mM SAM, 1 mM sodium dithionite, and varied amounts of CGA (0.025–800 *µ*M) were mixed in 50 mM HEPES buffer (pH 8.0). A 500-*µ*L reaction volume was used for the lower substrate concentrations to facilitate HPLC analysis. The reaction was incubated at 25°C, and aliquots were removed at appropriate time points for HPLC analysis as described above. The percent conversion as determined by HPLC was used to calculate the product’s quantity. The product for a given substrate concentration at different time points was plotted, and the initial rate was then plotted against substrate concentration. The results were fitted to the Michaelis–Menten equation by nonlinear regression using GraphPad Prism 5 to determine the *k*_cat_ and *K*_m_ values.

## Supporting Information

Figure S1 **Continuous-wave X-band EPR of BlsE under various conditions with or without glycerol.** A, As-isolated sample of BlsE (120* μ*M). B, Reconstituted BlsE (with and without glycerol) reduced by dithionite before loading into an EPR tube anaerobically. C, B with 1 mM SAM added to the mixture. D, B with 1 mM CGA added to the mixture.
(PDF)

Figure S2 **Two steps chemical synthesis of 8.**
(PDF)

Figure S3 **^1^H NMR (500 MHz) spectrum of compound 16.**
(PDF)

Figure S4 **^1^H NMR (500 MHz) spectrum of compound 8.**
(PDF)

Figure S5 **^1^H NMR (500 MHz) spectrum of compound 9.**
(PDF)

Figure S6 **^13^C NMR (125 MHz) spectrum of compound 9.**
(PDF)

Figure S7 **^1^H–^1^H COSY spectrum of compound 9.**
(PDF)

Figure S8 **HSQC spectrum of compound 9.**
(PDF)

Figure S9 **HMBC spectrum of compound 9.**
(PDF)

Figure S10 **HR-ESI-MS spectra of compounds 9.**
(PDF)

Figure S11 **HR-ESI-MS spectra of compounds 11.**
(PDF)

Figure S12 **SDS-PAGE results of mutant proteins.**
(PDF)

Table S1 **Primers used in this study.**
(PDF)
