# Supplementary figures and images for: Discovery and Characterization of BlsE, a Radical S-Adenosyl-L-methionine Decarboxylase Involved in the Blasticidin S Biosynthetic Pathway
Source: PLoS One. 2013 Jul 18;8(7):e68545. doi: 10.1371/journal.pone.0068545 (PMC3715490; doi:10.1371/journal.pone.0068545)

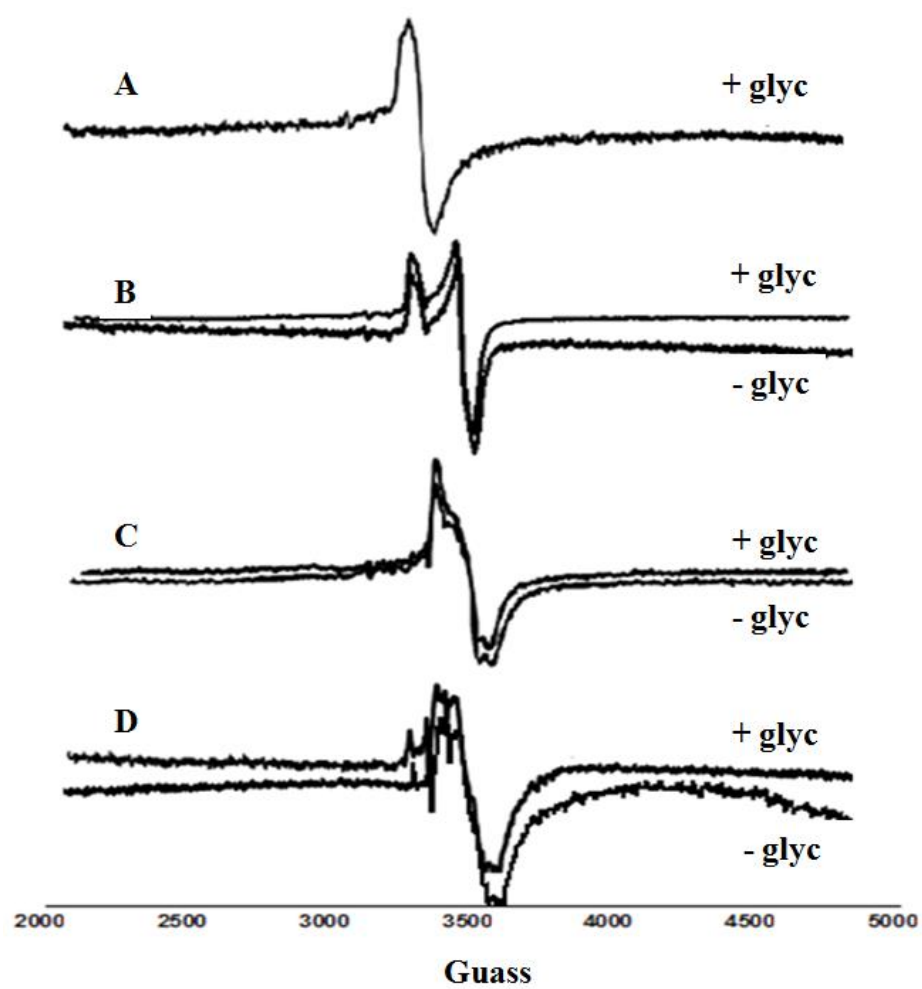

Supplement: Figure S1 — Continuous-wave X-band EPR of BlsE under various conditions with or without glycerol. A, As-isolated sample of BlsE (120 μM). B, Reconstituted BlsE (with and without glycerol) reduced by dithionite before loading into an EPR tube anaerobically. C, B with 1 mM SAM added to the mixture. D, B with 1 mM CGA added to the mixture. (PDF) [file pone.0068545.s001.pdf]

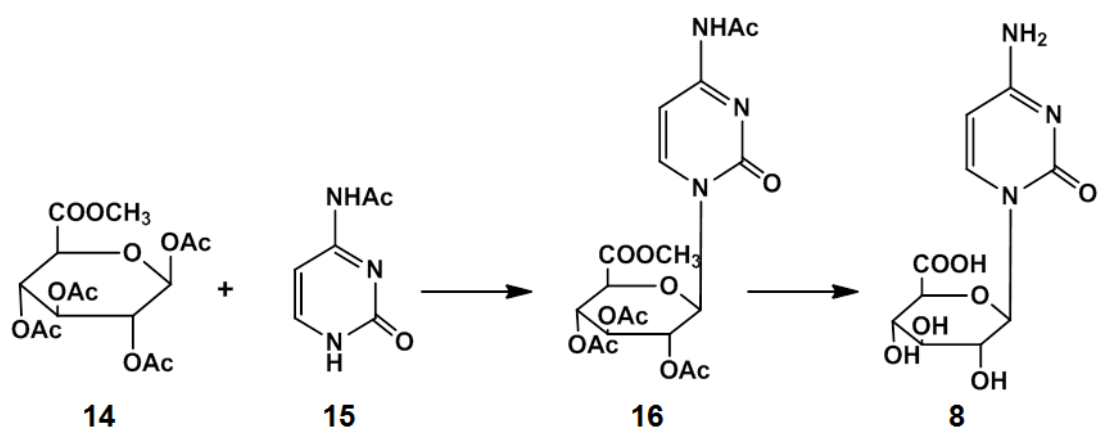

Supplement: Figure S2 — Two steps chemical synthesis of 8. (PDF) [file pone.0068545.s002.pdf]

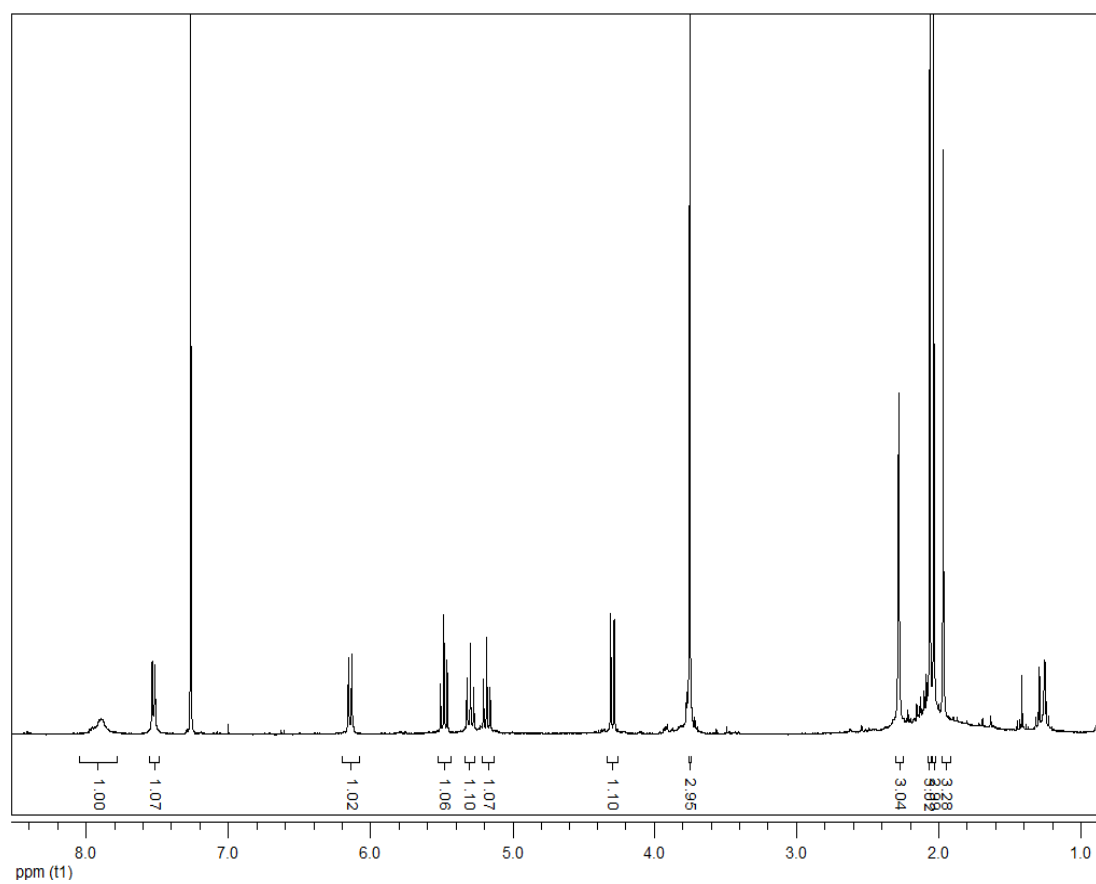

Supplement: Figure S3 — 1H NMR (500 MHz) spectrum of compound 16. (PDF) [file pone.0068545.s003.pdf]

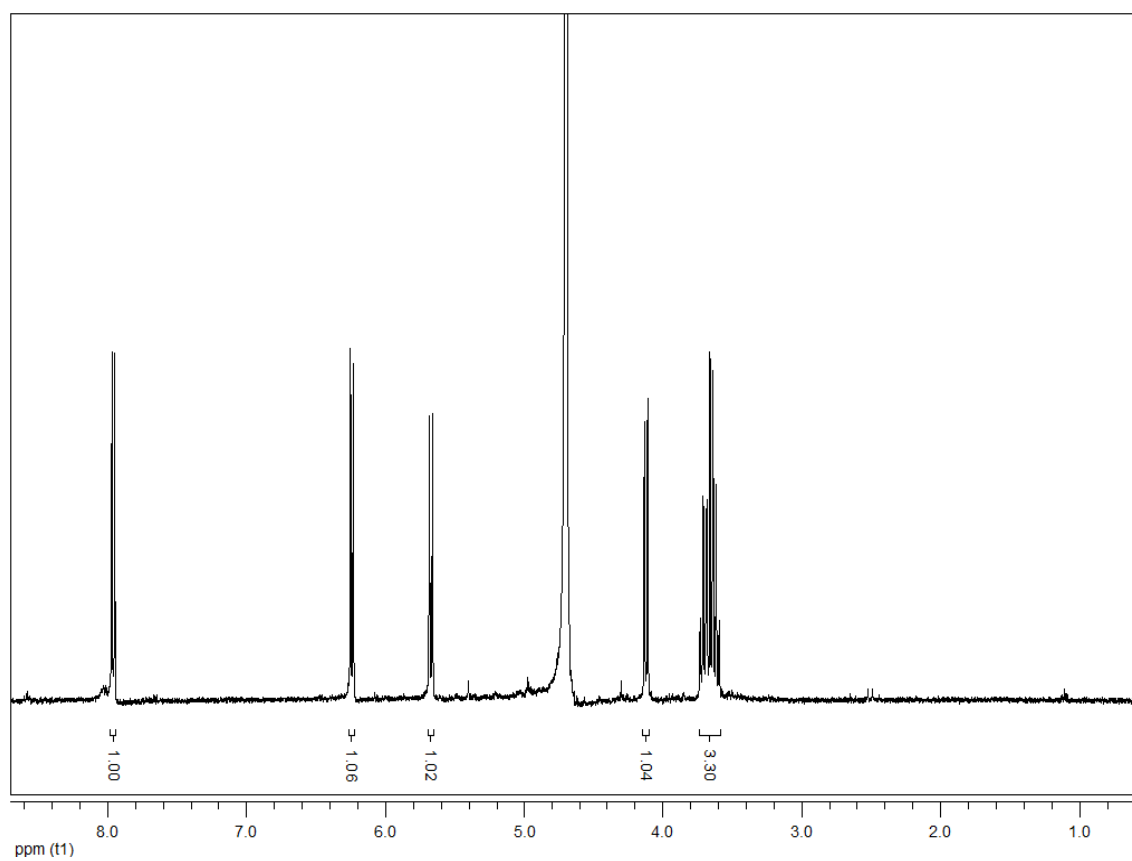

Supplement: Figure S4 — 1H NMR (500 MHz) spectrum of compound 8. (PDF) [file pone.0068545.s004.pdf]

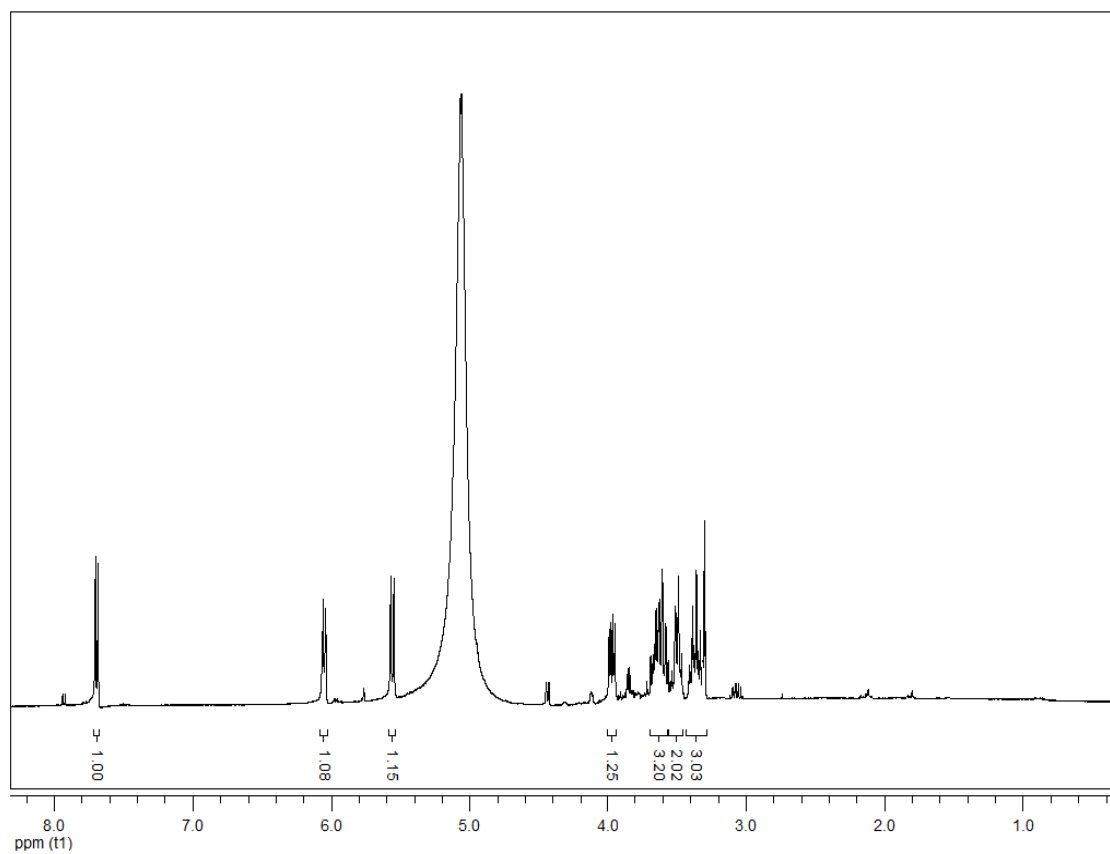

Supplement: Figure S5 — 1H NMR (500 MHz) spectrum of compound 9. (PDF) [file pone.0068545.s005.pdf]

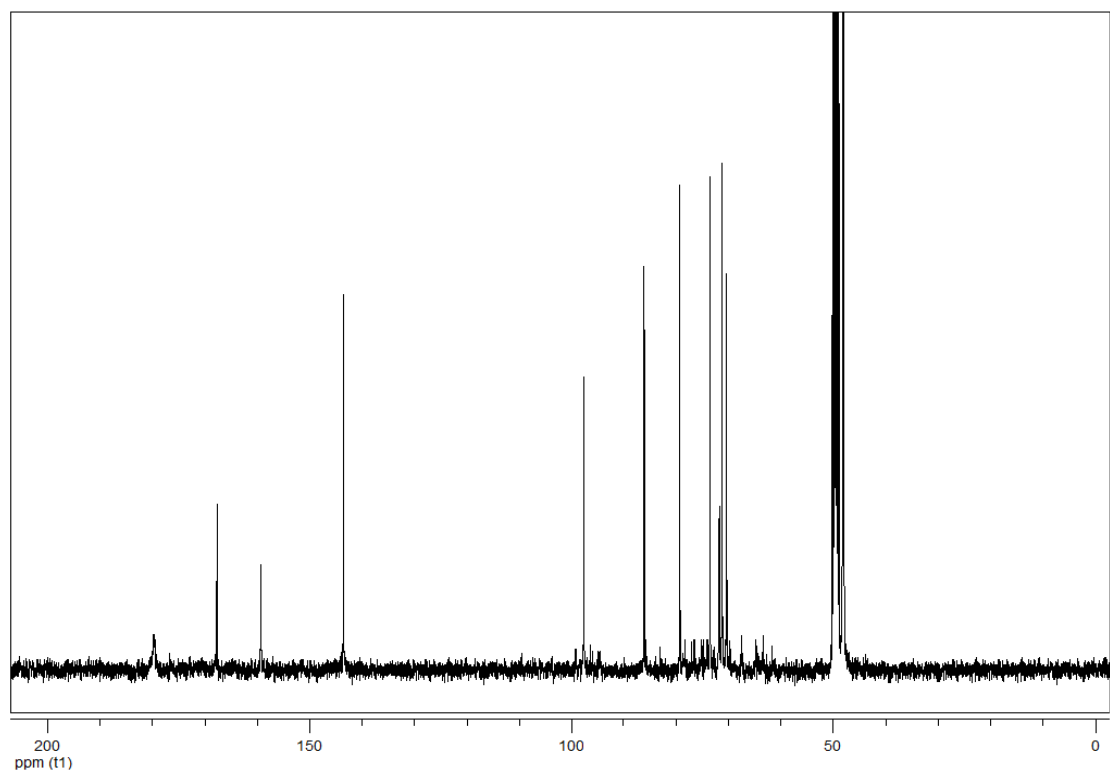

Supplement: Figure S6 — 13C NMR (125 MHz) spectrum of compound 9. (PDF) [file pone.0068545.s006.pdf]

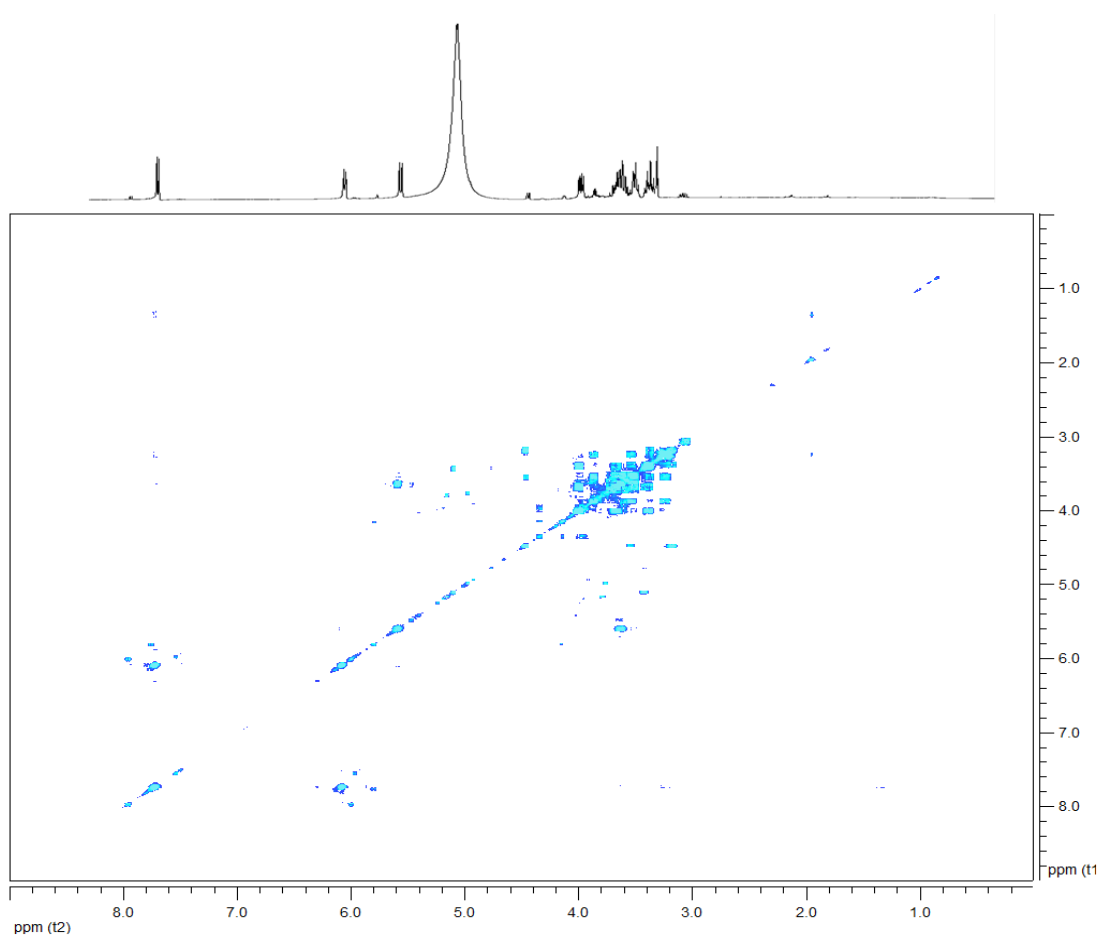

Supplement: Figure S7 — 1H–1H COSY spectrum of compound 9. (PDF) [file pone.0068545.s007.pdf]

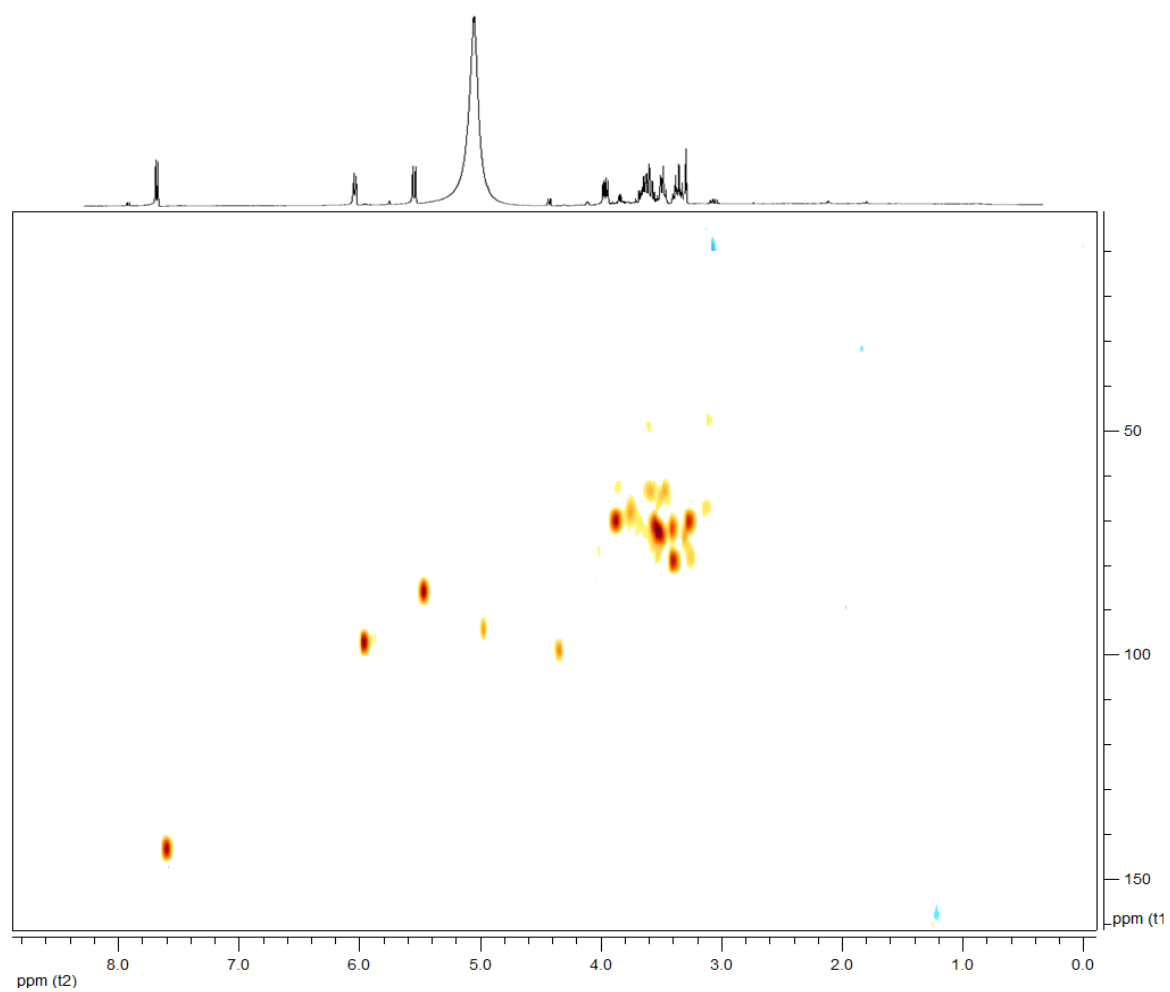

Supplement: Figure S8 — HSQC spectrum of compound 9. (PDF) [file pone.0068545.s008.pdf]

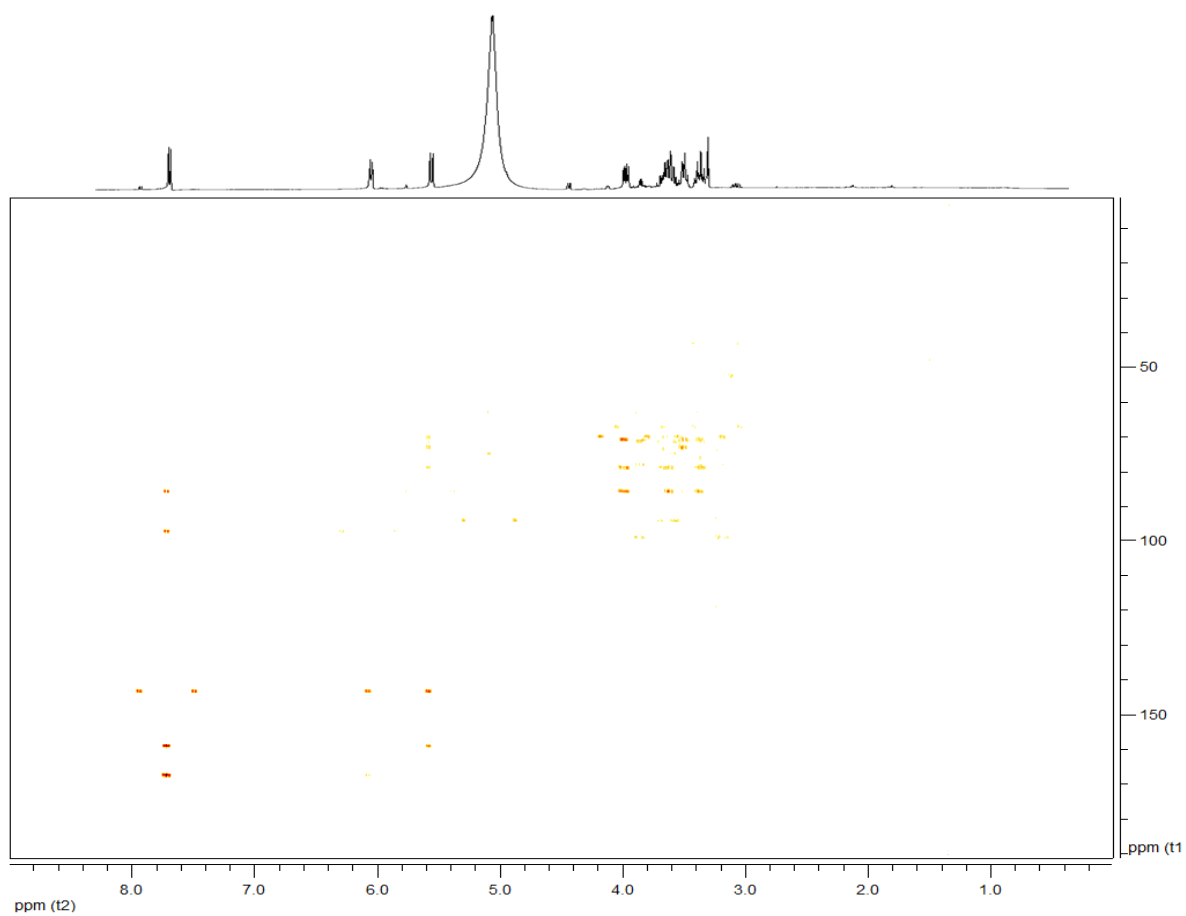

Supplement: Figure S9 — HMBC spectrum of compound 9. (PDF) [file pone.0068545.s009.pdf]

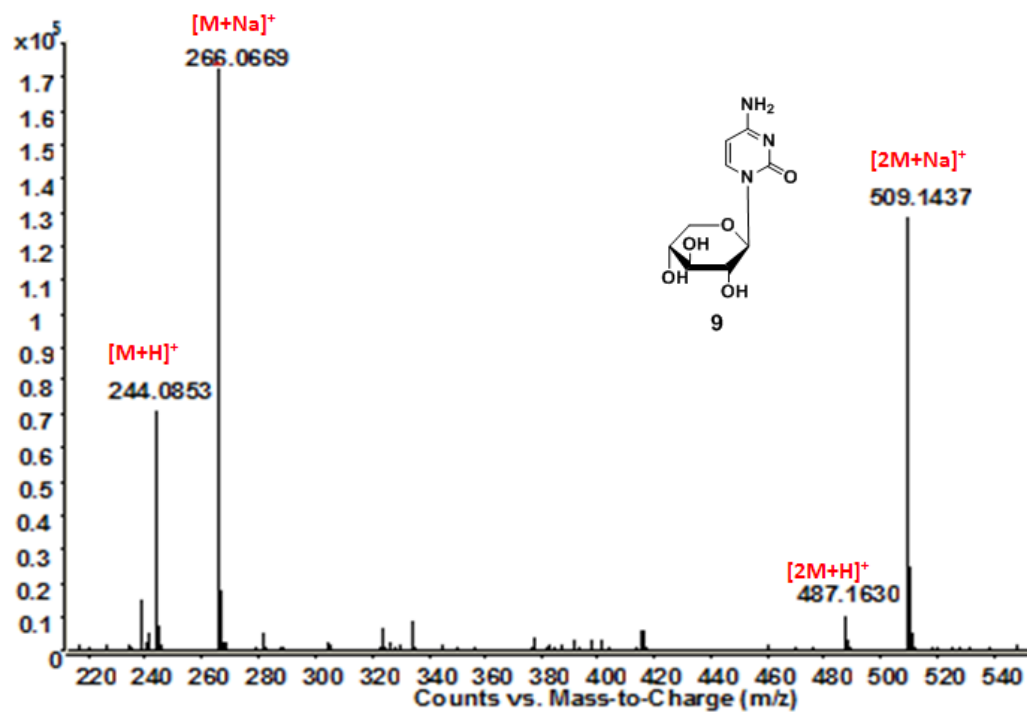

Supplement: Figure S10 — HR-ESI-MS spectra of compounds 9. (PDF) [file pone.0068545.s010.pdf]

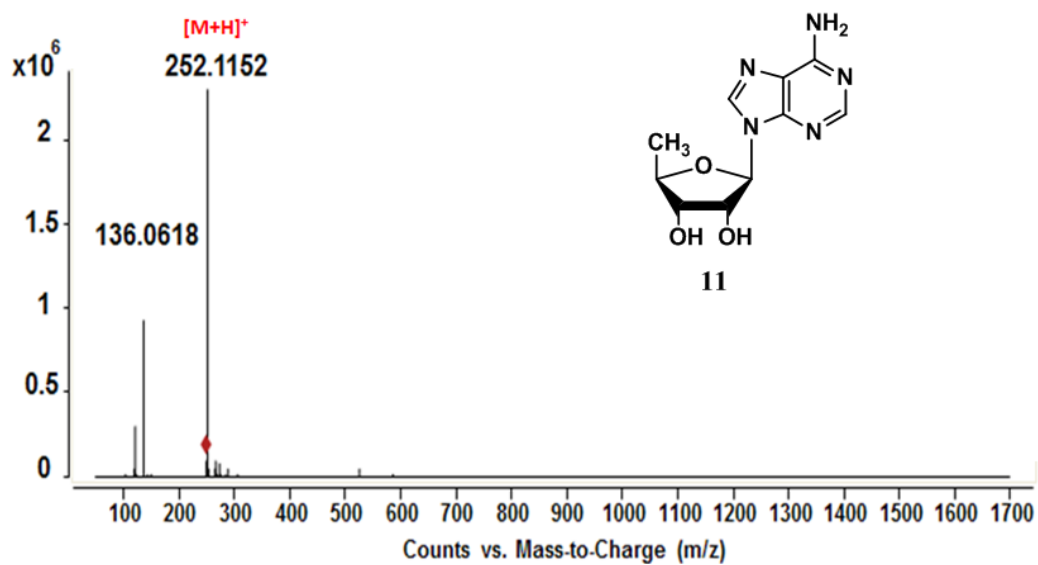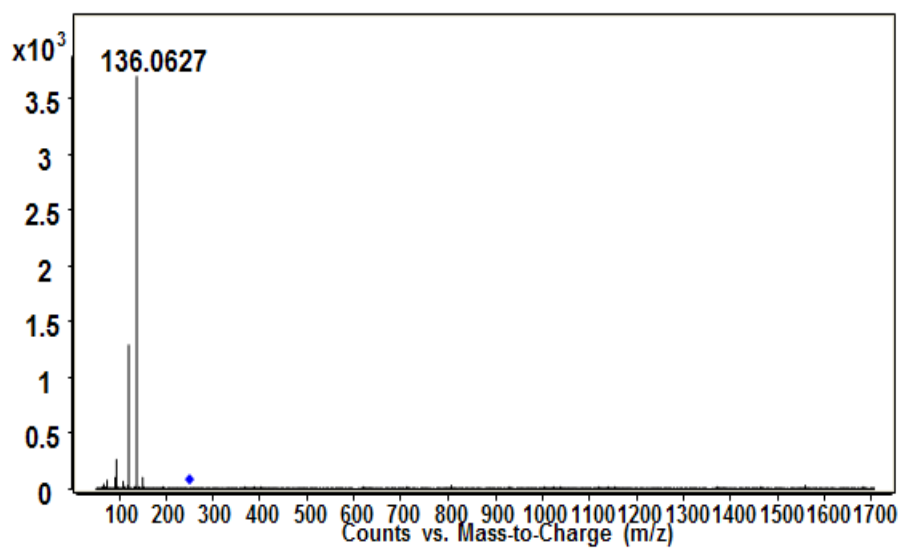

Supplement: Figure S11 — HR-ESI-MS spectra of compounds 11. (PDF) [file pone.0068545.s011.pdf]

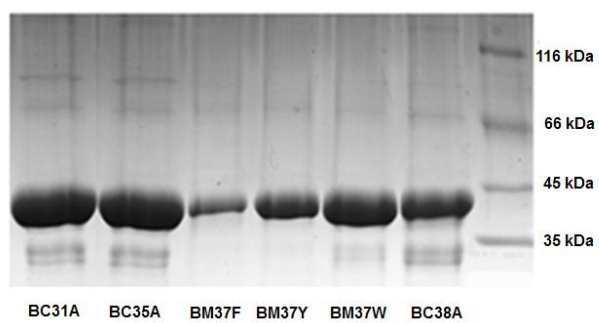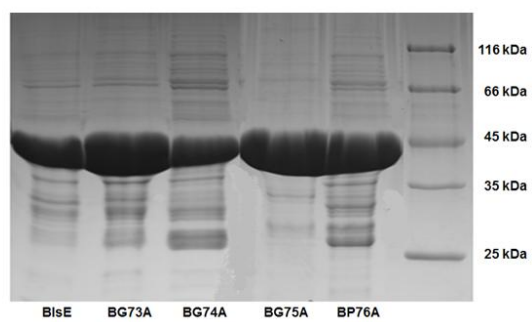

Supplement: Figure S12 — SDS-PAGE results of mutant proteins. (PDF) [file pone.0068545.s012.pdf]
