# Supplementary material for: Discovery and Characterization of BlsE, a Radical S-Adenosyl-L-methionine Decarboxylase Involved in the Blasticidin S Biosynthetic Pathway
Source: PLoS One. 2013 Jul 18;8(7):e68545. doi: 10.1371/journal.pone.0068545 (PMC3715490; doi:10.1371/journal.pone.0068545)
Supplement: Table S1 — Primers used in this study. (PDF) [file pone.0068545.s013.pdf]

**Primer code**	**Sequences of primers (5'-3')**
BlsE-For	AA***CATATG***ACCGAGCGAACGGCGTCCCG
BlsE-Rev	***GAATTC***CGCCTTGACCGATGGGGACCCGG
Fpr-For	AA***CATATG***GCTGATTGGGTAACAGGCAAAG
Fpr-Rev	***GAATTC***TGATGCCGGCATATCGCGGGATG
Fld-For	AA***CATATG***GCTATCACTGGCATCTTTTTC
Fld-Rev	***GAATTC***TATTAATACGGCGTAGACATG
BlsEC31A-For	GCCGCCAACGCGGACTGCTTCATGTGCGACTTC
BlsEC31A-Rev	CTCCAGCAGGCGGATGAACAGATACCTCTCGCG
BlsEC35A-For	GACGCCTTCATGTGCGACTTCGCACTCTCCCGG
BlsEC35A-Rev	CGCGTTGCAGGCCTCCAGCAGGCGGATGAACAG
BlsEM37F-For	TTCTGCGACTTCGCACTCTCCCGGGACACGTTC
BlsEM37F-Rev	GAAGCAGTCCGCGTTGCAGGCCTCCAGCAGGCG
BlsEM37Y-For	TACTGCGACTTCGCACTCTCCCGGGACACGTTC
BlsEM37Y-Rev	GAAGCAGTCCGCGTTGCAGGCCTCCAGCAGGCG
BlsEM37W-For	TGGTGCGACTTCGCACTCTCCCGGGACACGTTC
BlsEM37W-Rev	GAAGCAGTCCGCGTTGCAGGCCTCCAGCAGGCG
BlsEC38A-For	ATGGCCGACTTCGCACTCTCCCGGGACACGTTC
BlsEC38A-Rev	GAAGCAGTCCGCGTTGCAGGCCTCCAGCAGGCG
BlsEG73A-For	GCCGGCGAGCCGCTGATGCACACGGACGTGGCG
BlsEG73A-Rev	GGTGAAGCGGATGTAGCCGACCCCGGCCTCCAC
BlsEG74A-For	GCCGAGCCGCTGATGCACACGGACGTGGCGGAG
BlsEG74A-Rev	ACCGGTGAAGCGGATGTAGCCGACCCCGGCCTC
BlsEE75A-For	GCCCCGCTGATGCACACGGACGTGGCGGAGCTG
BlsEE75A-Rev	GCCACCGGTGAAGCGGATGTAGCCGACCCCGGC
BlsEP76A-For	GCCCTGATGCACACGGACGTGGCGGAGCTGGTG
BlsEP76A-Rev	CTCGCCACCGGTGAAGCGGATGTAGCCGACCCC
